# Acute myocardial infarction due to giant coronary artery aneurysm and arteriovenous fistula: a challenging case report and review of the literature

**DOI:** 10.1186/s12872-024-03851-w

**Published:** 2024-04-01

**Authors:** A. Vassilikou, MP Xenitopoulou, K. Ziampa, AP Evangeliou, S. Mitsiadis, A. Syrnioti, G. Petrakis, P. Tossios, V. Vassilikos, S. Tzikas

**Affiliations:** 1 3rd Department of Cardiology, “Hippokration” General Hospital of Thessaloniki, Aristotle University of Thessaloniki, Thessaloniki, Greece; 2 Pathology Department, “AHEPA” University Hospital of Thessaloniki, Aristotle University of Thessaloniki, Thessaloniki, Greece; 3 Cardiothoracic Surgery Department, “Hippokration” General Hospital of Thessaloniki, Aristotle University of Thessaloniki, Thessaloniki, Greece

**Keywords:** Coronary artery aneurysm, Coronary artery fistula, Giant coronary artery aneurysm, Myocardial infarction, NSTEMI, Case report

## Abstract

**Background:**

A coronary artery aneurysm (CAA) is an abnormal dilation of a coronary artery segment often accompanied by coronary artery fistula (CAF), leading to communication between a coronary artery and a cardiac chamber or a part of the coronary venous system. Both CAAs and CAFs can present with symptoms and signs of myocardial ischemia and infarction.

**Case presentation:**

We describe the case of a 46-year-old woman with non-ST-elevation myocardial infarction (NSTEMI) caused by a “giant” CAA. Various imaging modalities revealed a thrombus-containing aneurysm located at the right-posterior cardiac border, with established arteriovenous communication with the distal part of left circumflex artery (LCx). After initial treatment with dual antiplatelet therapy, a relapse of pain was reported along with a new increase in troponin levels, electrocardiographic abnormalities, reduced left ventricular ejection fraction (LVEF) and thrombus enlargement. Surgical excision of the aneurysm was favored, revealing its true size of 6 cm in diameter. Τhe aneurysm was excised without complications. The patient remained asymptomatic during follow-up.

**Conclusions:**

Management of rare entities such as “giant” CAAs and CAFs can be challenging. Cases such as this can serve as precedents to facilitate treatment plans and develop consistent recommendations, emphasizing the importance of personalized strategies for future patients.

**Supplementary Information:**

The online version contains supplementary material available at 10.1186/s12872-024-03851-w.

## Background

Both CAAs and CAFs are typically discovered incidentally during coronary angiography or noninvasive cardiac imaging. However, in this case, the CAA presented as an NSTEMI event.

A CAA is defined as an abnormal dilation of a coronary artery segment exceeding the diameter of normal adjacent segments or the patient’s largest coronary vessel by 1,5 times [1]. The most common cause of CAA is atherosclerosis, responsible for over 90% of the cases in adults, while other causes include congenital malformations (17%), Kawasaki vasculitis (17%) in children, Takayasu arteritis, connective tissue disorders and infections, with mycotic aneurysms being accountable for 11% of the cases [2]. CAA formation also seems to be the result of stent implantation during percutaneous coronary intervention (PCI) [3]. A “giant” CAA is defined by a diameter of more than 20 mm and is often accompanied by CAFs [4].

A CAF is a significant malformation of the coronary arteries defined as the abnormal communication between a coronary artery and a cardiac chamber or a part of the coronary venous system. The true incidence of CAF is speculative but ranges between 0,4 − 1,2% among different studies [5]. Approximately 10% of CAFs can cause a coronary artery aneurysm due to volume overload leading to remarkable dilatation of the coronary artery [6, 7]. While the majority of CAFs are congenital, they can also result from cardiac surgery, intracardiac device implantation, myocardial biopsy and direct trauma [8]. CAFs commonly originate from the right coronary artery (RCA) and drain into the right ventricle (RV) [9]. Fistulous communication with aneurysmal dilatation between the LCx and the coronary sinus is a rare phenomenon, with prevalence rates of 18.3% and 7% respectively [9].

Here we present a symptomatic case of a “giant” LCx artery aneurysm with a fistula draining into the coronary sinus. Despite its usual discovery being incidental, this case highlights the manifestation of “giant” CAA as an NSTEMI event. From our research, only 125 cases have been mentioned in the literature using the keywords “acute myocardial infarction AND giant coronary artery aneurysm” in the PubMed database. We highlight the dynamic presentation and not only a static morphology of the aneurysm while adding interesting histologic findings from the tissue excised. Due to their rarity and the general inability to conduct large clinical trials on cases like this, we strongly believe that our case contributes additional evidence, providing useful and educative insights into the personalized management of “giant” CAAs.

## Case presentation

A 46-year-old female presented to the emergency department with intermittent chest pain radiating to the back and the left subclavian area starting 8 h prior. She had a smoking history of 20 pack years and a positive cardiac family history. The rest of the personal history was insignificant. The physical examination was unremarkable. The initial 12-lead electrocardiogram did not show any abnormal findings; however, laboratory results revealed an increased troponin value, indicating myocardial damage. Thus, the patient was admitted to the coronary care unit, with suspected NSTEMI and a coronary angiography was planned. The pharmacological treatment plan included dual antiplatelet therapy with aspirin and clopidogrel, atorvastatin and omeprazole. Interestingly, bedside echocardiography revealed a structurally and functionally normal heart, except for a cystic formation 25 mm in diameter and low flow gradient, located in the infero-lateral cardiac wall.

**Fig. 1:**
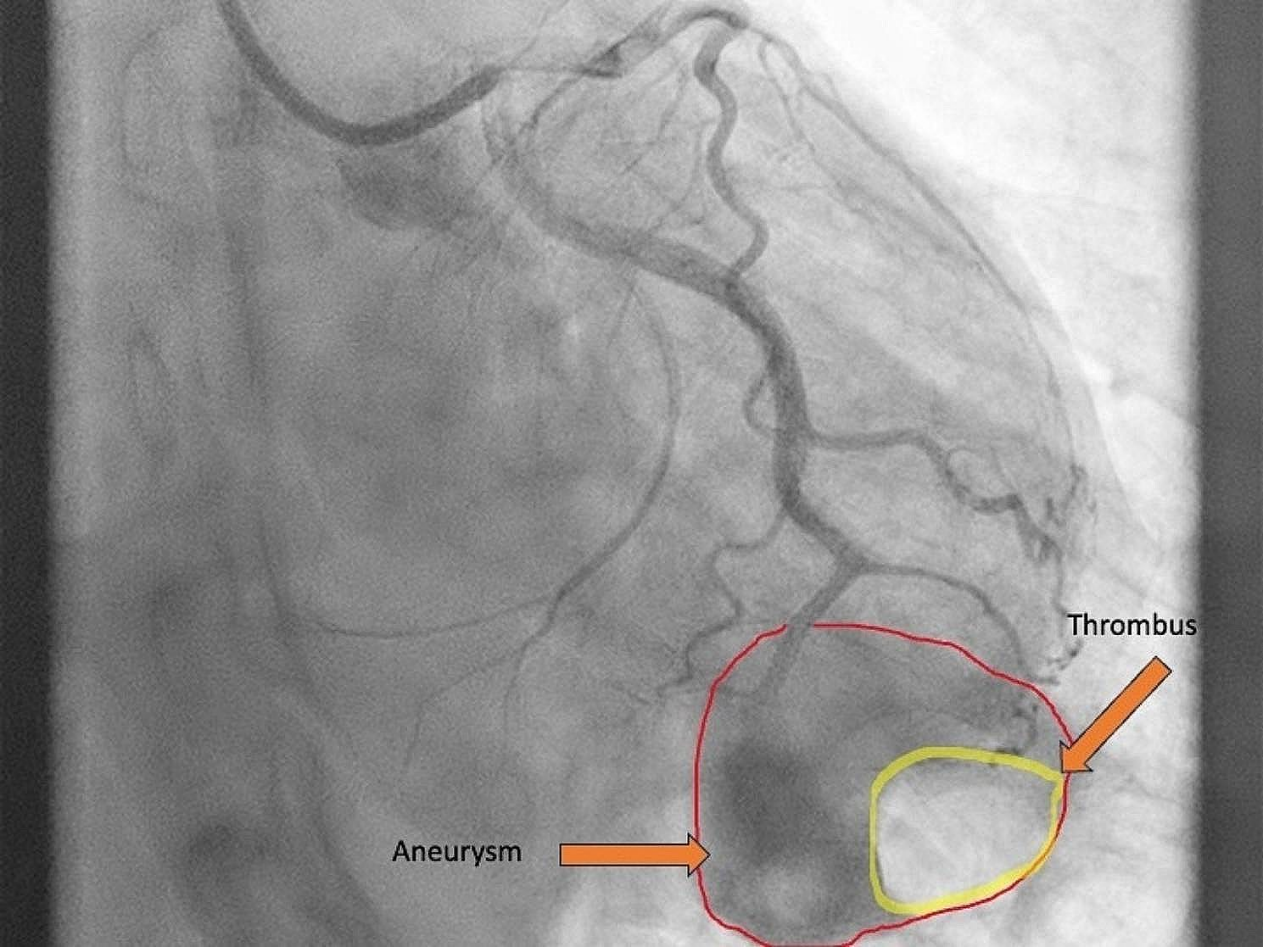
Coronary angiography presenting the aneurysm at the distal part of the LCx.

Coronary angiography performed the following day revealed a coronary aneurysm with arteriovenous communication and thrombus formation without stenosis in the periphery of the LCx in the absence of coronary artery disease (Fig. 1, supplementary movie 1, supplementary movie 2).

**Fig. 2:**
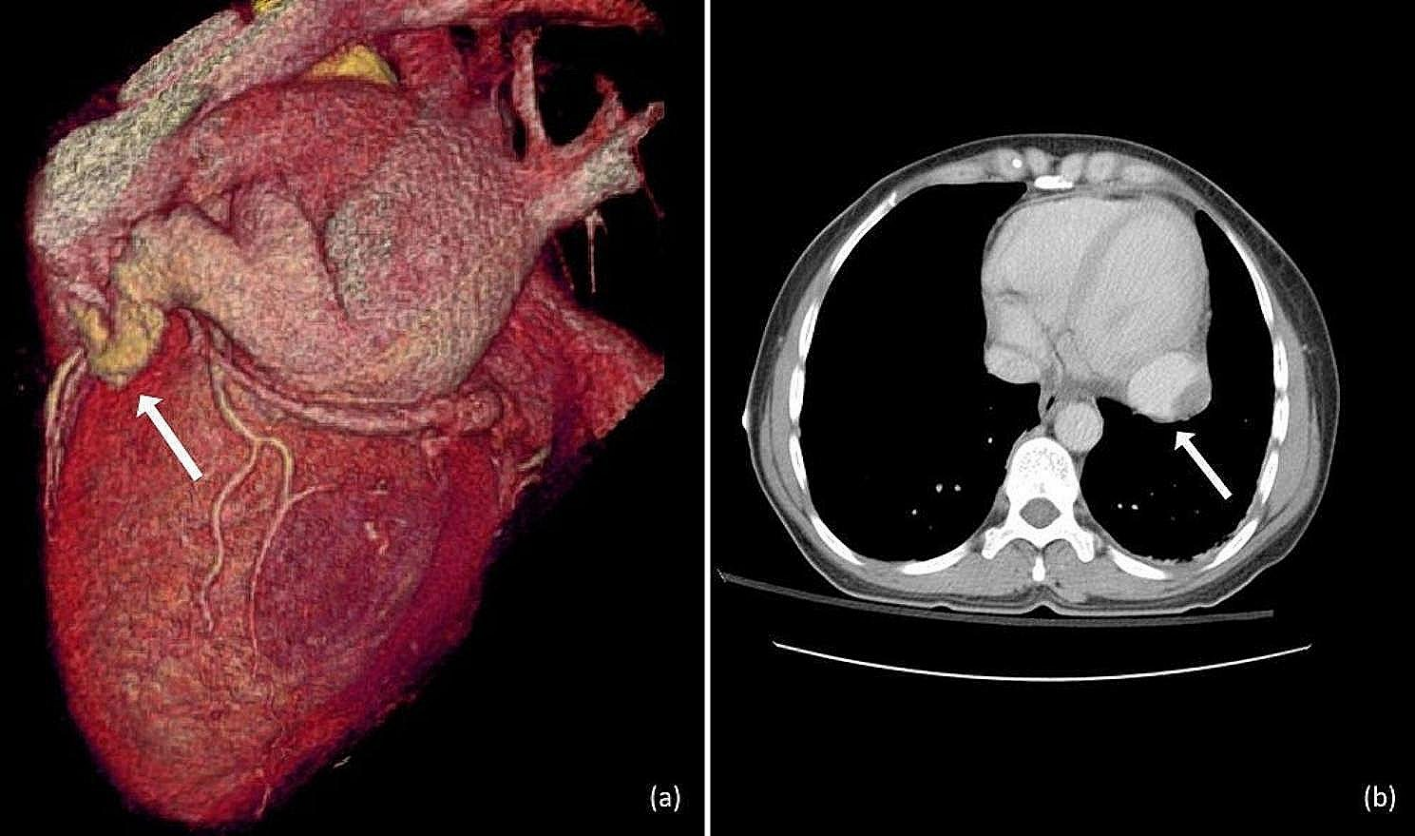
3D reconstruction (**a**) and depiction of the thrombus in the aneurysm through Computed Tomography (**b**)

Cardiac computed tomography (CCT) revealed the presence of an aneurysm of 4,3 cm diameter located at the right-posterior cardiac border with thrombus formation (∼ 13 mm diameter) within the aneurysmal sac and an arteriovenous communication with the distal part of the LCx (Fig. 2).

**Fig. 3:**
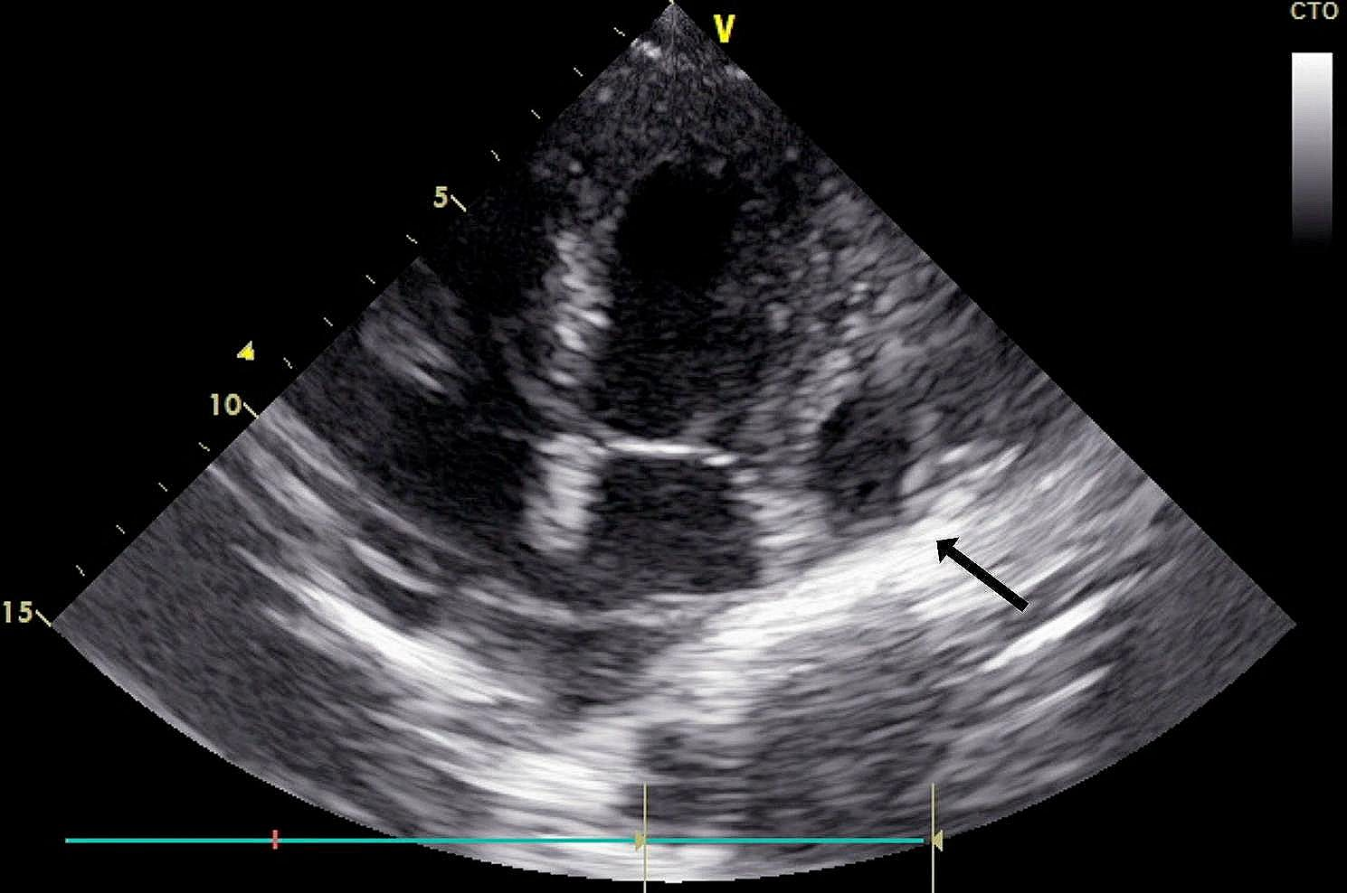
Echocardiographic apical 4 chamber view of the patient’s heart depicting the aneurysm lateral to the mitral valve

The following day, the patient reported pain relapse accompanied by a new increase in troponin levels and electrocardiographic abnormalities in the inferolateral wall (negative T waves in leads II, III, aVF and V5-V6). Repeated echocardiography revealed a marginally reduced LVEF of 50–55% with hypokinesia of the posterior cardiac wall and right ventricle. Additionally, a hyperechoic area was observed in the aneurysmal space consistent with thrombus enlargement (Fig. 3, supplementary movie 3).

After consultation with the hospital’s cardiac surgeons, surgical excision of the aneurysm was favored. The aneurysm was macroscopically visible after elevation of the heart apex, measuring approximately 6 cm. The aneurysm was excised revealing the thrombus. Following the removal of the thrombus, the proximal and distal orifices were revealed and sutured. The aneurysm was ligated, and the patient was weaned off the Cardiopulmonary bypass (CPB) without complications.

**Fig. 4:**
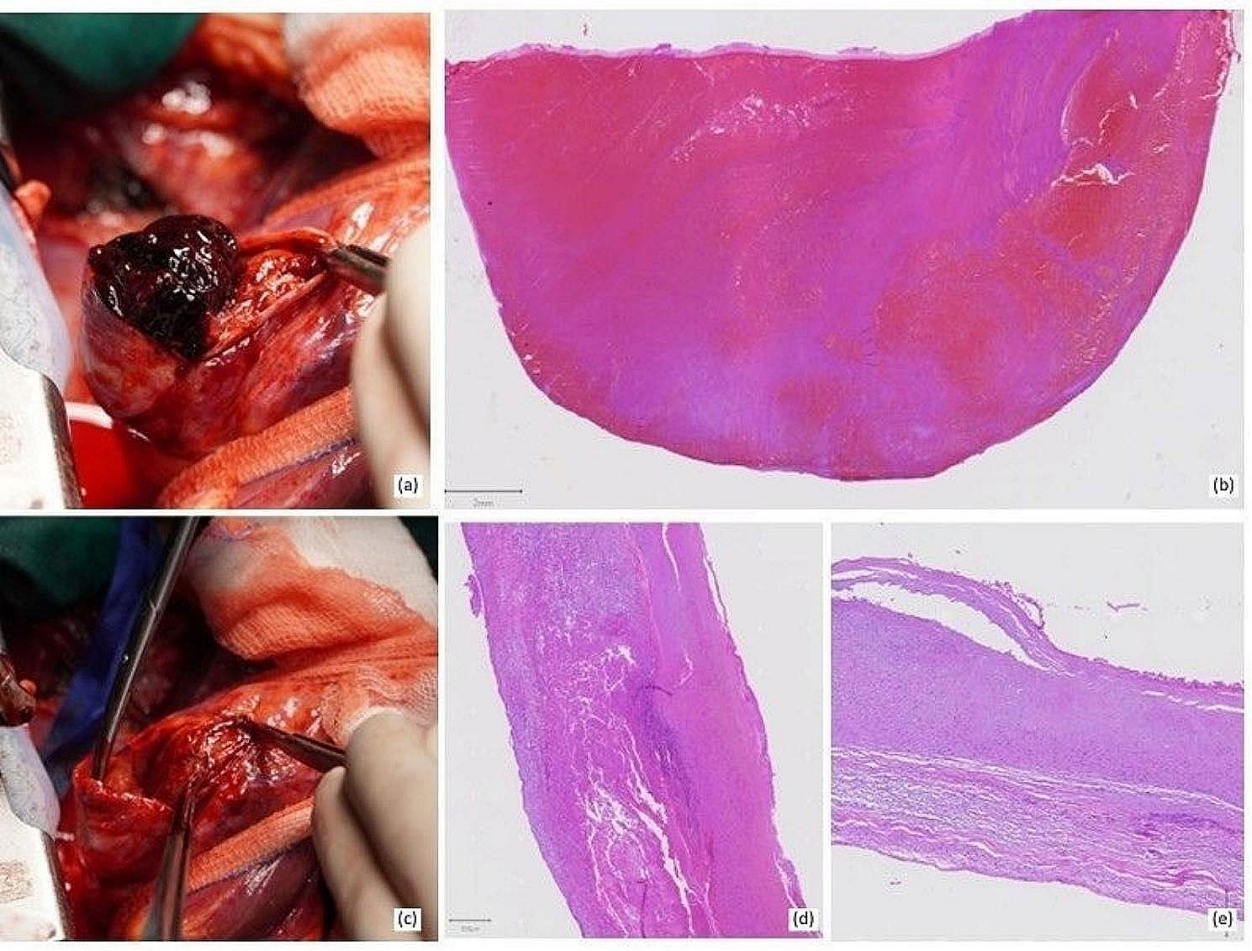
Macroscopic (**a, c**) and microscopic (**b, d, e**) view of the coronary aneurysm with arteriovenous communication and thrombus formation

Histopathological examination of the aneurysmatic tissue revealed an intima with mild myxomatoid changes, while the media was characterized by disarray of the smooth muscle and the elastic fibers. These findings seem to have resulted in the structural weakness of the vascular wall contributing to the formation of the sac [10]. There was evident separative destruction of the media wall with thrombus material found inside similar in constitution to the separate pieces of the large thrombus, with abundant erythrocytes, fibrin deposition and inflammatory cells. There were no findings of atherosclerotic lesions (Fig. 4). The histopathological examination did not show any specific findings towards vasculitis or connective tissue disorder. Therefore, the most probable cause of the CAA is supposed to be a congenital malformation.

The patient was discharged with the recommendation for 12 months dual antiplatelet therapy according to acute coronary syndrome (ACS) guidelines. During follow-up, the patient remained asymptomatic, with normalization of both the LVEF and the electrocardiogram.

## Discussion

Both CAAs and CAFs can present with angina, dyspnoea, and signs of myocardial ischemia and infarction. Angina is thought to result from the coronary steal phenomenon, which is related to the diastolic pressure gradient caused by the runoff from a high-pressure coronary artery to a low resistance receiving cavity, thus making the myocardium distal to the origin of the fistula and prone to ischaemia. Moreover, the left-to-right shunt contributes to the development of angina and ischemia [9]. CAFs draining into the major coronary veins of the right heart chambers have similar effects in the circulation as an atrial septal defect [11]. Untreated CAFs can cause life-threatening complications, including thrombosis, cardiac tamponade and myocardial ischaemia and infarction [12]. Coronary aneurysms present with chest pain, dyspnea, paroxysmal nocturnal dyspnea, palpitations and fatigue [1]. Complications of CAA are rare but can be fatal and include thrombosis, distal embolization of the vessel, rupture, vasospasm, heart failure and cardiac compression or cardiac tamponade [13–15].

Coronary angiography is the imaging modality of choice. This technique provides information on the anatomy of CAAs and CAFs and the entire coronary artery structure. However, the true aneurysm size cannot be accurately measured in case of intramural thrombi. Additional imaging modalities supporting the diagnosis include multi-slice coronary artery computed tomography (MSCT-CA), transthoracic (TTE) and transesophageal echocardiography (TEE). A combination of multiple imaging modalities can be utilized to define the entire pathway of the CAF, which is crucial for preprocedural planning [16].

Treatment options for CAFs include transcatheter closure, cardiac surgery, or conservative treatment. The choice of intervention depends on various factors such as the size and anatomy of the fistula, its distal or proximal location, the presence of symptoms or complications related to the fistula, the patient’s age and their co-morbidities [11]. A heart team approach is crucial in decision-making and evaluating CAFs [17]. Conservative management is considered the best option in asymptomatic individuals, small fistulas, and patients with various comorbidities unable to undergo surgery, for whom regular-follow up is recommended. Small CAFs can close spontaneously over time [18], whereas medium or large sized CAFs tend to enlarge [19]. Indications for CAF closure include ischaemia in the territory of the affected coronary artery, arrhythmic events possibly related to the CAF, endarteritis, vessel rupture, cardiac chamber enlargement and ventricular dysfunction [20]. There are no consistent guideline recommendations due to variable size, anatomic and physiologic variants. The first transcatheter closure of a CAF was performed in 1983 by Reidy et al. [21]. Various techniques and approaches, including transarterial or transvenous approaches as well as the use of coils, vascular plugs or covered stents, have been developed [20]. The approach and materials depend on the criteria mentioned and the physician’s expertise. The goal is to eliminate the flow of the fistula and isolate all the feeders while preserving myocardial viability. Complications, such as device migration and occlusion, vascular trauma and residual flow have been reported in the literature [20].

Surgery is the usual treatment for CAFs due to their complex and often tortuous anatomy. It is indicated in large CAFs characterized by aneurysmal dilation and thrombus formation. Median sternotomy is used for access. CPB with or without cardioplegia is often employed for proper macroscopic visualization and safe dissection of the CAF. CAF closure can be achieved through the epicardium or the endocardium or with suture ligation [22]. A surgical closure of the CAF has a reported mortality rate of less than 1% [11]. Complications following surgery include periprocedural ST segment changes, myocardial infarction, stroke, pneumothorax and arrhythmias. Incomplete closure and residual flow have been reported in approximately 10% of the patients [11, 22]. Postprocedural anticoagulation is recommended for safe and good long-term outcomes.

Surgery options for treating CAA include coronary artery bypass grafting (CABG), resection or ligation of the CAA [23].

In our patient, surgical excision was favored due to local expertise and the concern that embolization coils might further migrate to the distal end of the fistulous aneurysm and finally land in the venous circulation due to the size of the aneurysm.

## Conclusions

Despite their rarity, both CAAs and CAFs pose evident challenges for both interventional cardiologists and cardiac surgeons. The broad spectrum of clinical manifestations and the anatomical complexity of the malformations make the management of such cases highly personalized. Considering parameters such as the hemodynamics of the affected area and the various coexisting complications, detailed reports of the approach used for each specific case of CAA and CAF are imperative. Our case report highlights a “giant” CAA located at the right-posterior cardiac border, presenting as an NSTEMI event, which is not a typical presentation. Emphasizing the need for the bypass surgery pathway for thrombus-containing CAAs, characterized by an arteriovenous communication with the distal part of LCx we aim to ensure better outcomes for similar cases. Ultimately, we hope this report contributes to the enrichment of a database aimed at the development of consistent guidelines over the challenging and uncommon cardiological entities as this one.

### Electronic supplementary material

Below is the link to the electronic supplementary material.

Supplementary Material 1

Supplementary Material 2

Supplementary Material 3

## Data Availability

The datasets used and/or analysed during the current study available from the corresponding author on reasonable request.

## Abbreviations

CAA: Coronary Artery Aneurysm
CAF: Coronary Artery Fistula
NSTEMI: Non-ST-Elevation Myocardial Infarction
LCx: Left Circumflex Artery
LVEF: Left Ventricular Ejection Fraction
PCI: Percutaneous Coronary Intervention
RCA: Right Coronary Artery
RV: Right Ventricle
CCT: Cardiac Computed Tomography
CPB: Cardiopulmonary Bypass
ACS: Acute Coronary Syndrome
MSCT-CA: Multi-Slice Coronary Artery Computed Tomography
TTE: Transthoracic Echocardiography
TEE: Transesophageal Echocardiography
CABG: Coronary Artery Bypass Grafting
